# Investigating an effort avoidance account of attentional strategy choice

**DOI:** 10.3758/s13414-024-02927-1

**Published:** 2024-07-26

**Authors:** Tianyu Zhang, Andrew B. Leber

**Affiliations:** Department of Psychology, The Ohio State University, 225 Psychology Building, 1835 Neil Avenue, Columbus, OH 43210 USA

**Keywords:** Attentional control, Visual search, Strategy, Cognitive effort, Demand selection

## Abstract

People often choose suboptimal attentional control strategies during visual search. This has been at least partially attributed to the avoidance of the cognitive effort associated with the optimal strategy, but aspects of the task triggering such avoidance remain unclear. Here, we attempted to measure effort avoidance of an isolated task component to assess whether this component might drive suboptimal behavior. We adopted a modified version of the Adaptive Choice Visual Search (ACVS), a task designed to measure people’s visual search strategies. To perform optimally, participants must make a numerosity judgment—estimating and comparing two color sets—before they can advantageously search through the less numerous of the two. If participants skip the numerosity judgment step, they can still perform accurately, albeit substantially more slowly. To study whether effort associated with performing the optional numerosity judgment could be an obstacle to optimal performance, we created a variant of the demand selection task to quantify the avoidance of numerosity judgment effort. Results revealed a robust avoidance of the numerosity judgment, offering a potential explanation for why individuals choose suboptimal strategies in the ACVS task. Nevertheless, we did not find a significant relationship between individual numerosity judgment avoidance and ACVS optimality, and we discussed potential reasons for this lack of an observed relationship. Altogether, our results showed that the effort avoidance for specific subcomponents of a visual search task can be probed and linked to overall strategy choices.

**Supplementary information:**

The online version contains supplementary material available at 10.3758/s13414-024-02927-1.

## Introduction

Humans have a powerful and flexible attentional control system to deal with various tasks in our daily lives. This attentional control system can be conceived of as having two important facets: the strategy of selecting which control settings to apply and the ability to actually implement the control settings (Irons & Leber, 2018a, 2020). Though we may have a wide range of abilities, ability alone does not guarantee optimal performance in a given task. This is because you must know when—and be willing—to apply the abilities that are most suited to current behavioral demands. For instance, biasing your attention to a specific color may help you find your orange car when it is parked among black and white cars, but the same strategy will be inefficient when you want to find your friend, who is wearing the same colored shirt as most of the crowd (e.g., at a sporting event). Thus, it is essential to measure the impact of strategy on task performance. We define strategy as a mental plan or policy that guides individuals on how to prioritize and use selected sensory information (Leber & Irons, 2019; Liesefeld et al., 2023). We are particularly interested in the degree to which people optimize their strategy. As illustrated in the previously mentioned example, the optimality of a strategy depends on the individual’s goal and the current task environment. In a scenario of a visual search task, we define the optimal strategy as the one yielding the best performance—specifically that which will result in the fastest search without sacrificing accuracy. Previous studies have shown evidence that the choice of strategy plays an important role in determining visual search performance (Boot et al., 2009; Irons & Leber, 2016; Kristjánsson et al., 2014; Nowakowska et al., 2017).

Researchers have long questioned why participants would perform suboptimally (Bacon & Egeth, 1994). While it has been argued that people tend towards optimal visual search (Najemnik & Geisler, 2005, 2009), several demonstrations of suboptimality have been reported. In one such study, Boot and colleagues (2009) found that people often made unnecessary eye movements while performing a change detection task, which was detrimental to reaction time (RT). Similarly, Nowakowska and colleagues (2017) reported that participants performing visual search for an oriented bar target tended to fixate on locations that could have otherwise been searched covertly. These unnecessary fixations significantly slowed RT.

To systematically study strategy, Irons and Leber (2016, 2018a) designed the Adaptive Choice Visual Search (ACVS) task, which can specifically measure the optimality of people’s visual search strategies on a trial-by-trial basis. In what we call the “standard” version of this task (Irons & Leber, 2018a, Experiment 2), participants view a search display composed of red, blue, and green squares, each containing a digit from 2 to 9. On each trial, two target digits are presented, one in the red color subset and the other in the blue color subset. These targets always contain the digits 2, 3, 4, or 5, although the two targets never contain the same digit. Participants can choose to search for either target, as only one target needs to be reported. Critically, on each trial, one color subset is less numerous than the other. Thus, the optimal strategy is to search through the color subset containing fewer items to find the target within that set. The target in the smaller subset is considered the optimal target because only about half as many items need to be searched compared with the large subset. As such, our primary measure of strategy use is the percentage of trials in which individuals report the target from the smaller subset (i.e., the optimal target; we refer to this dependent measure as the *optimality rate,* or simply *optimality*).^1^ Irons and colleagues have typically found participants to be far from optimal in their target choices, with mean optimality typically not exceeding 70% (e.g., Irons & Leber, 2018a); additionally, they have observed notable individual variation in this metric, which has been shown to be stable when assessed with test–retest reliability and internal consistency measures (see Irons & Leber, 2020).

Why do people frequently adopt suboptimal attentional strategies? There are several possibilities. Much like in everyday visual searches, the ACVS requires multiple interconnected steps to achieve optimal performance. For instance, one must appraise the display (Hansen et al., 2019) and then evaluate and determine the most effective attentional control strategy within the current environment before commencing a search for the target (Cain et al., 2012; O’Leary & Sloutsky, 2017; Wolfe, 2013). Moreover, in such a changing environment, optimizing responses requires sustained proactive control to maintain the task goal and choose the best strategy (Braver, 2012; Hansen et al., 2019), which further increases cognitive demands (Braver et al., 2007; Locke & Braver, 2008). Failing to perform any of these component steps adequately may result in suboptimal performance.

As for why someone might fail to perform one of these steps, here we consider that people may be avoiding cognitive effort. Irons and Leber (2018a) provided initial evidence in support of a general effort avoidance hypothesis. In that study, people who reported the optimal strategy to be more subjectively effortful were less likely to use it. Note that participants were only asked to provide subjective ratings of effort at the end of each task block. Such a survey probe carries limitations. First, only one data point was collected per participant; second, such ratings are retrospective and may not reflect the effort experienced at the moment the strategy selections are made. More relevant to the present work, the results of Irons and Leber did not link effort avoidance with any particular subcomponent of the ACVS task.

In the present work, we sought evidence for effort avoidance within subcomponents of the ACVS. As mentioned above, multiple potential task components could be involved in achieving optimal performance. The avoidance of any related effort of these task components could result in suboptimal behavior (see Kool et al., 2010; Shenhav et al., 2017). Previous studies have highlighted the essential role of environmental appraisal, especially the numerosity judgment process when determining which color subset contains fewer items. In one study, we found that participants became more optimal when extra time was provided to view the display of colored squares only (Zhang et al., 2024). Furthermore, participants’ optimality decreased significantly when the numerosity judgment process was interrupted (Hansen et al., 2019). Others have also reported that enumeration or counting tasks required more effort investment than search tasks (Porter et al., 2007). In line with all these findings, we theorized that the suboptimal behaviors in the ACVS are at least partially driven by the avoidance of cognitive effort related to appraising the display to identify the optimal color subset (the color subset with a smaller number of squares), which we refer to as numerosity judgment effort.

Previous studies (Ariely, 2001; Chong & Treisman, 2003) have shown that people can extract statistical information, such as mean set size, from a display rapidly and accurately, which might suggest that the enumeration process is relatively effortless. However, promptly utilizing this extracted information to make judgments and guide subsequent actions may indeed require cognitive effort. We propose that individuals may indeed harbor a tendency to avoid such numerosity judgments. This inclination can become particularly pronounced when the numerosity judgment task is embedded into a set of more complex and dynamic task demands. To test this hypothesis, we measured whether people would demonstrate some degree of avoidance of task stages relating to estimating and comparing the color subsets. Further, we investigated whether people’s degree of effort avoidance can predict attentional strategy optimization during the ACVS task. Additionally, we also measured people’s numerosity judgment ability to test whether it could predict attentional strategy optimalization or avoidance of numerosity judgment demand.

In Experiment 1, we sought to assess avoidance of numerosity judgment. We further questioned how such avoidance might relate to visual search strategy choice. We adopted the demand selection task (DST) from Kool and colleagues (2010) to measure people’s effort avoidance relating to the ACVS task. To specifically isolate the elements of estimating and comparing color subsets, we designed a new word-cue search task with two types. One provides a numerosity cue while the other provides a color cue. The stimulus is very similar to the one used in the ACVS task, but with an additional word cue presented at the center of the screen. On the one hand, a numerosity cue could be “large” or “small. The large cue informs participants that the target will appear in the more numerous color subset; the small cue informs them that it would be in the less numerous color subset. On the other hand, a color cue could be “red” or “blue.” This indicates which color subset participants must search to find the target. The critical difference between these two cue types is the requirement to estimate and compare the color subsets in the numerosity condition. No such process is required in the color cue condition, in which participants can immediately begin to search through the cued color subset.

This additional step required in the numerosity versus color cue mirrors the key difference in performing the ACVS optimally or not. That is, it is necessary to optionally take on this additional step in order to achieve optimal performance. Our logic in this study is that the additional step may be avoided during the ACVS due to its effort requirements. We seek evidence in support of this effort hypothesis by placing the numerosity and color cue tasks into a demand selection procedure.

The experiment was divided into three parts. In the first part, we use the ACVS task to measure participants’ visual search strategies. In the second part, we measured numerosity judgment ability via a task with similar stimulus parameters to the ACVS, in which participants judged which of two colored sets of squares was less numerous. In the final part, we used the demand selection task mentioned above to measure individuals’ avoidance of numerosity judgment effort.

If the avoidance of numerosity judgment effort is a key factor in determining visual search strategy selection in the ACVS, we can make two predictions. First, at the group level, participants should prefer the color cue task over the subset cue task during the demand selection. Second, on an individual level, participants who show greater avoidance of the subset cue task during demand selection should show lower rates of optimality in the ACVS. Additionally, we tested whether numerosity judgment ability is a key factor in determining visual search strategy selection in ACVS. If numerosity judgment ability predicts strategy choice, then greater performance at the numerosity judgment task will be linked to higher optimality rates in the ACVS.

## Experiment 1

### Method

Experiment 1 was preregistered on the Open Science Framework (OSF). Detailed information regarding the rationale, methods, and planned analyses can be found in the OSF preregistration file (https://osf.io/6nbhq). Note that we found one oversight in our preregistration plan, in which we neglected to declare how we would test demand avoiding (or seeking) in the demand selection task. Following Kool et al. (2010) and Westbrook et al. (2013), we used the Wilcoxon signed-rank test to conduct this analysis.

#### Participants

Fifty individuals (24 men, 23 women, one nonbinary, two unreported) aged 18–41 years were included in Experiment 1. All participants were recruited through the Prolific platform and were paid $9.60 for roughly 1 hour of participation. All participants were prescreened by their locations (USA), their Prolific approval rate (≥96%), submission number (≥50), and all self-reported normal or correct-to-normal visual acuity. All participants were provided with the online informed consent form approved by The Ohio State University institutional review board.

Based on the preregistered exclusion criteria, an additional 74 participants were excluded from further analysis. This was due to (1) low accuracy on the ACVS task (accuracy lower than 80% or more than three standard deviations below the group average; *N* = 20) and (2) low accuracy on the numerosity judgment task trials with the ratio of 1:2 (accuracy lower than 90% or more than three standard deviations below the group average; *N* = 54).

Given the high exclusion rate, our predetermined exclusion criteria appear to have been too stringent for this online sample, particularly for the numerosity judgment task. Thus, in addition to the analysis stipulated by our preregistration plan, we also include exploratory analysis with a relaxed accuracy cutoff, in which data from 25 of these participants are included (self-reported gender: 10 men, 15 women).

#### Stimuli and procedure

This study was conducted online, with participants using their own computers, so the screens on which the stimuli were presented varied in size and viewing distance. Thus, we cannot report visual angle degrees. Instead, we report stimulus sizes in a fixed visual angle unit. This unit was created such that, with a typical 24-in. display with a viewing distance of 68 cm, 1 unit = 1º visual angle. The experiment consisted of three types of tasks, as follows.

##### Adaptive Choice Visual Search

The stimuli were based on previous studies using the ACVS. Since several variants of the ACVS have been developed, it is useful to declare the specific version we used here, which we describe as the “standard ACVS with preview.” What we describe as the standard version was first presented by Irons and Leber (2018a, Experiment 2), and the preview is described by Hansen et al. (2019) and Zhang et al. (2024).

**Fig. 1:**
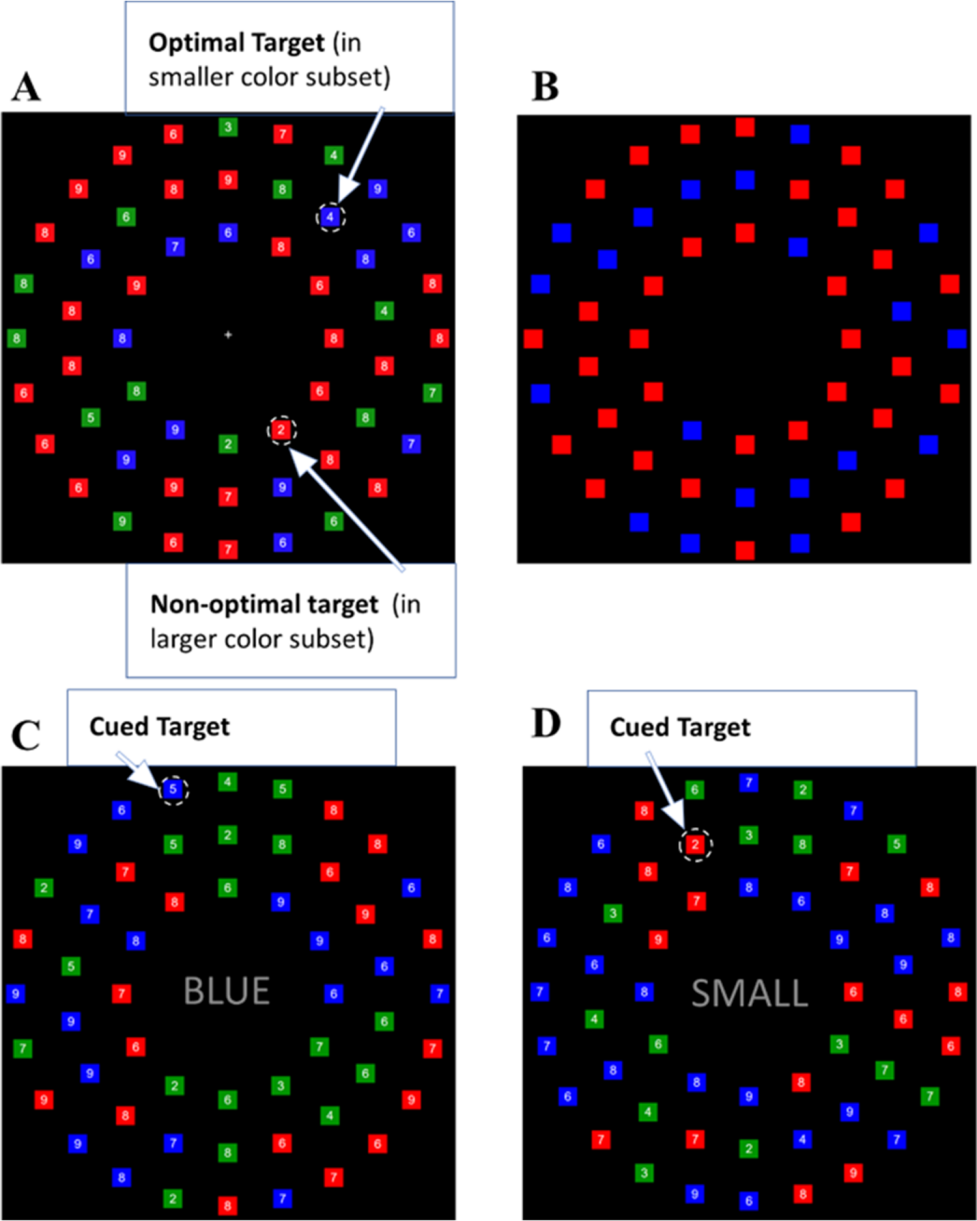
**A** An example of the ACVS task. Two targets are highlighted. The one in the smaller color subset (blue in this case) is considered more optimal to search for than the other one in the larger color subset (red) because there are fewer items to search through. **B** An example of the numerosity judgment task. Participants need to indicate the color of the smaller subset (blue in this case). **C** An example of the color cue condition of the Word-Cue Search task. The blue 5 is the target since the blue subset is cued. **D** An example of the numerosity cue condition of the word-cue search task. The red 2 is the target since the small subset is red. (Color figure online)

On each search trial, the display contained 54 colored squares (sized 1*1 unit) with 13 red squares, 13 blue squares, 14 green squares, and 14 variably colored squares. The color of the variable squares alternated between all red and all blue for runs of one to six successive trials. All colored squares were randomly arranged in three concentric rings around a fixation cross at the center of the screen. The radii of the inner, middle, and outer rings were 5.6, 8.5, and 11.3 units, respectively. The number of colored squares in the inner, middle, and outer rings were 12, 18, and 24, respectively, each spaced equidistantly within their respective rings. Each colored square contained a white digit (0.65 units, font: Arial) placed at the center of the square. Two digits randomly picked from the set [2, 3, 4, 5] without replacement served as targets on each search trial, with one in the red set and one in the blue set. For all other red and blue squares, digits from the set [6, 7, 8, 9] were randomly assigned. Green squares contained digits randomly chosen from 2 to 9; the inclusion of all digits served to dissuade participants from ignoring color and adopting a deliberate search for the target digits (see Fig. 1A).

For every search trial, the fixation cross was first displayed at the center of the screen for 400 ms, followed by a 1,000-ms preview containing colored squares only without any digits. We have begun to incorporate preview displays in this task to give participants more time to appraise the displays prior to commencing search, which can boost optimality (Zhang et al., 2024). This is especially useful for online study samples, who have tended to produce lower optimality rates (McKinney et al., 2020). After the preview, the digits were added to the squares, at which point search for the target digits could commence. Participants were instructed that two targets were present on every trial and that they only needed to find one on each trial. They were always free to search for either target on each trial. Upon finding a target, participants identified the digit inside the target by pressing the corresponding key on the keyboard (V, B, N, or M corresponding to 2, 3, 4, or 5, respectively). Once a response was registered, the stimuli were removed from the screen, and feedback was provided for 1,000 ms to indicate whether the response was correct or not. If the response was incorrect, a 400-Hz auditory tone was also played for 150 ms during the feedback. Before the new trial began, there was an intertrial interval of 500 ms. Participants first completed a 10-trial practice block, followed by two blocks of the ACVS task with 84 trials per block. Between each block, participants could take self-paced breaks.

##### Numerosity judgment task

The stimuli for this numerosity judgment task were like the ACVS task. However, displays only contained 54 red and blue squares with different ratios and no green squares, and digits were not presented (see Fig. 1B). In half of the trials, the smaller subset was colored red, while the other half of the smaller subset was colored blue. The stimuli were displayed for 500 ms and then replaced with a blank screen. Participants were asked to compare the number of blue squares versus red squares and report the color of the smaller subset by pressing the keys V (for red) and B (for blue). Response was allowed for up to 5 seconds, at which point the trial was declared an error. The order of trials was randomized in the way that one color would not be the target color (the color of the smaller subset) for more than six consecutive times. In this task, we had five ratio conditions (1:1.08, 1.25:1, 1.45:1, 1.7:1, 2:1), and each condition had 24 trials with a total of 120 trials. The order of different types of trials was randomized and interleaved.

##### **Word-cue search task**

The word-cue search contained both *color* and *numerosity* cue conditions. On each trial, participants were cued to search for the target in a specific subset of colored squares. The stimuli were similar to the ACVS, with a few key differences. First, the fixation cross was replaced by the word cue (text size 1.75 units, font: Arial). Second, the stimuli only contained one target, and the word cue specified the colored subset containing the target. For the color cue condition, “RED” or “BLUE” cues were presented at the center to indicate the relevant subset containing the target (see Fig. 1C). Similarly, for the numerosity cue condition, “LARGE” or “SMALL” cues were presented to indicate the target subset (always red or blue; see Fig. 1D). Here, red and blue subsets are equally likely to be large or small subsets. Thus, overall, the target set in both cued conditions was matched. In addition, as an extra layer of control, we included a digit between 2 and 5 that did not match the target in the uncued subset, which served as an additional distractor and ensured that participants would use the cue to achieve accurate performance. For each condition, participants first received five practice trials followed by a block of 40 trials of each cue type. This allowed participants to be familiar with both cued search conditions before the demand selection task.

##### **Demand selection task**

**Fig. 2:**
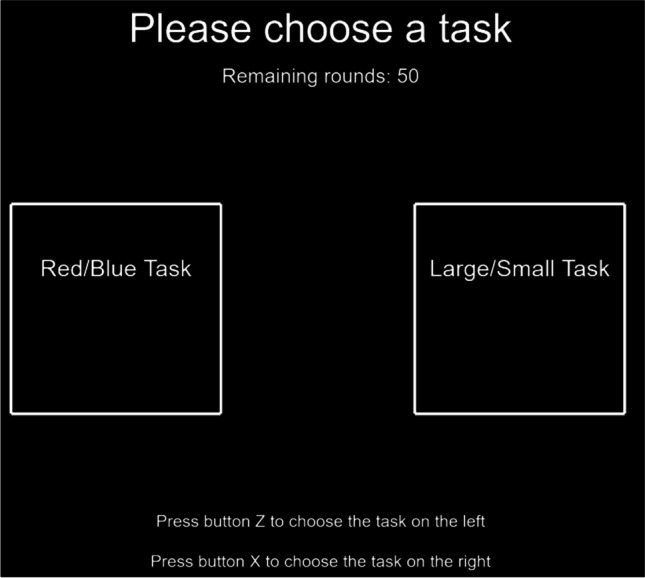
An example of the demand selection task with the name of two types of word-cue search task presented symmetrically on both sides of the screen

On each trial, the monitor displayed two white squares, presented symmetrically on both sides of the screen, with the name inside indicating the type of word-cue search task (see Fig. 2). The original mapping of task type to screen side was counterbalanced across participants. Participants were asked to use the keyboard to select one of the two tasks, pressing Z to select the task on the left and X to select the task on the right. After that, one trial of the selected word-cue search task was presented. The display was centered towards the side that the chosen task was presented on during the selection (deviated 6.3 units from the center), to reinforce the match between the selected and performed task. The task-location mapping was switched once, halfway through the selections, to disentangle preferences for the tasks from preferences for a specific button or side of the screen.

Note that, given the significant differences between the two search tasks, participants could easily discern the task type based on the provided word cue. Therefore, unlike the classic DST, which cued each task with abstract drawings (Kool et al., 2010), we provided explicit information about the task types during the selection procedure to prevent participants from overthinking the selection task and to ensure clarity (see Fig. 2). Consequently, our data primarily focused more on the choice rate after the detection of the demand difference, as discussed by Juvina et al. (2018), reflecting the pure avoidance of this numerosity judgment demand.

### Results and discussion

Mean accuracy for standard ACVS was 93.9%. In the analysis of response time (RT), we excluded trials in which participants made an incorrect response, as well as trials with RTs less than 300 or more than three standard deviations of the mean (3.56% of the standard ACVS trials).

#### ACVS

First, we calculated the main strategy measure reported by Irons and Leber (2016, 2018a), which was defined as the percentage of correct trials in which participants chose the target in the small colored subset. We refer to that target as the optimal target and the corresponding search strategy as the optimal strategy. Note that even if people are largely using the optimal strategy, they could occasionally notice and then opportunistically report the nonoptimal target (see Irons & Leber, 2016, 2018a). Notwithstanding, previous experiments have repeatedly revealed that searching for the target in the smaller color subset is the most efficient way of finding the target, as high optimality has been reliably associated with faster RTs (replicated in this experiment; see RT results below).

Results from the Standard ACVS revealed vast individual differences in optimality, ranging from .36 to .98 (*M* = .656, *SD* = .211). Mean RT was 2781 ms (range: 1568–4218, *SD* = 695). RT showed a significant relationship with optimality, *r*(48) = −.72, *p* < .001, indicating that the more optimal the overall search strategy, the faster RT in this task (see also Irons & Leber, 2018a; Li et al., 2022; McKinney et al., 2022; Zhang et al., 2024).

#### Numerosity judgment

To assess each participant’s ability to rapidly estimate and compare color subsets, we fitted a Weibull function to their accuracy rates across all five ratio conditions, plus one theoretical point (0.5,1) representing the chance level accuracy if the ratio had been set to 1:1. We defined the numerosity judgment ability as the threshold at which 75% accuracy was achieved on the fitted psychometric function. For instance, a score of 1.25 indicates the participant is estimated to achieve 75% accuracy at a 1.25:1 ratio. Results yielded a mean numerosity judgment ability of 1.28 (range: 1.07–1.69, *SD* = .14).

#### Cued search task

For the color cue condition, the mean accuracy was .949 (*SD* = .045) and the mean RT was 3,265 ms (*SD* = 578). For the subset cue condition, the mean accuracy was .879 (*SD* = .147) and the mean RT was 4,044 ms (*SD* = 713). Results revealed a significant difference between these two conditions in both accuracy, *t*(49) = 3.93, *p* < .001, and RTs, *t*(49) = 9.48, *p* < .001.

#### Demand selection task

**Fig. 3:**
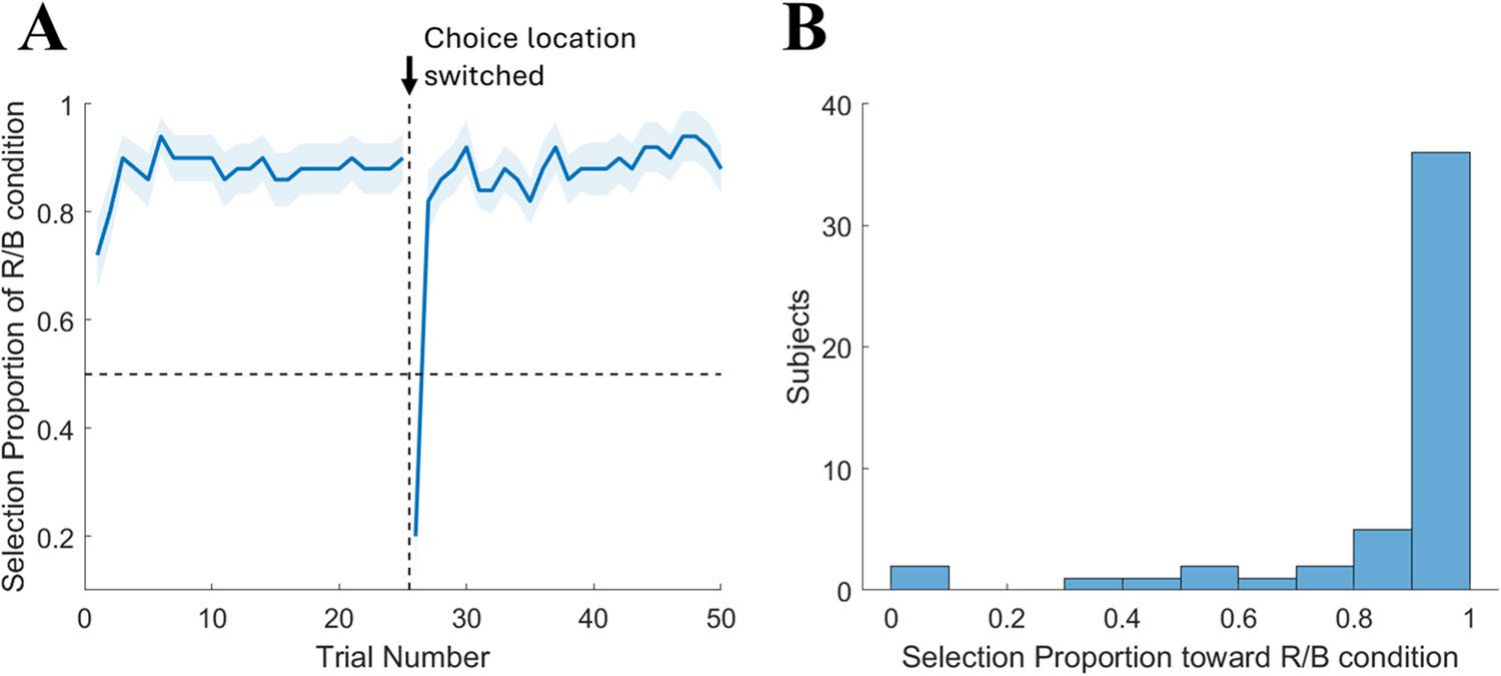
**A** Group-averaged proportion of the low-demand choice (red/blue), plotted by trial number, during the demand selection task in Experiment 1. **B** Histogram of individuals’ low-demand selection proportion in Experiment 1. R/B = red/blue color cue task

For the demand selection task, we assessed participants’ avoidance of the numerosity judgment task as the percentage choice rate of the low demand alternative (the color cue task) instead of the high-demand alternative (subset cue task). Across all participants, the average proportion of trials on which the low-demand alternative was selected was .867 (*SD* = .231). Forty-six participants (92%) chose the low-demand alternative more frequently than the high-demand alternative. The overall choice rates differed significantly from chance level (Wilcoxon signed-rank test, *p* < .001, effect size *r* = .769). The percentage rate of low demand choice over the course of 50 trials is shown in Fig. 3A. A histogram showing the distribution of the choice rates for each participant was presented in Fig. 3B. Overall, we observed a strong avoidance of the numerosity judgment task at the group level.

#### Relationships between measures

We compared how the task measures related to one another. We used traditional null hypothesis significance testing for all Pearson correlations, and we report Bayes factors to characterize relative support for the alternative versus null hypotheses. Bayes factors were computed using the correlationBF function in R package BayesFactor (Version 0.9.12-4.7; Morey & Rouder, 2024), using a noninformative prior (prior scale = ultrawide).

We first tested the relationship between numerosity judgment ability (75% threshold) and ACVS optimality rate, which was not significant, *r*(48) = −.14, *p* = .32; BF_10_ = 0.28. This null finding is consistent with our previous findings that cognitive abilities are largely independent of strategy (Irons & Leber, 2018a, 2020).

**Fig. 4:**
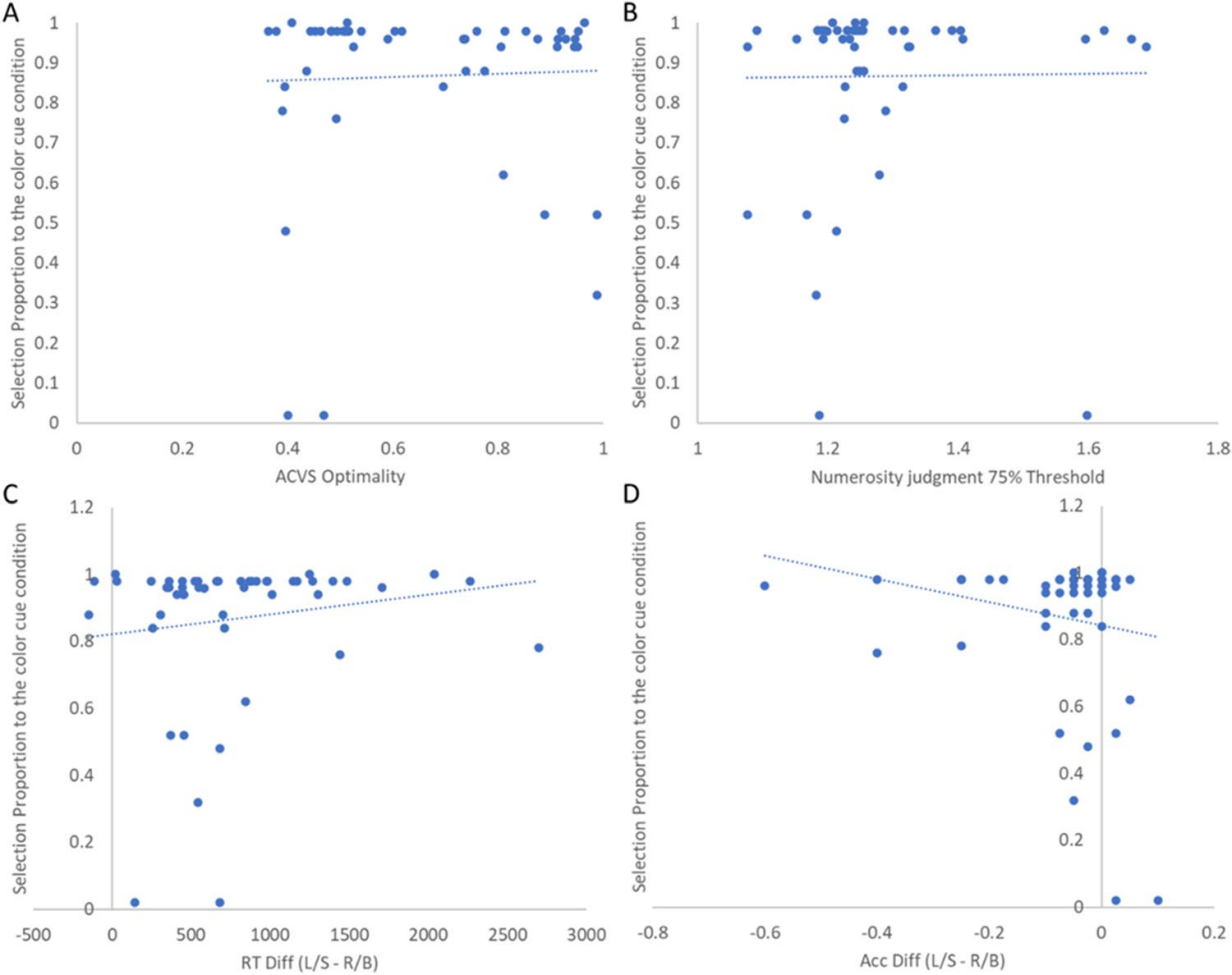
Scatter plots of correlations in Experiment 1 between (**A**) ACVS Optimality and DST selection proportion to the color cue task; (**B**) numerosity judgment ability and DST selection proportion; (**C**) RT differences between two types of word-cue search task and DST selection proportion; (**D**) accuracy differences between two types of word-cue search task and DST selection proportion

Next, we asked whether individuals’ demand selection rates predicted ACVS optimality. Specifically, does greater avoidance of the numerosity judgment predict poorer ACVS optimality? The correlation between these two variables was not significant, *r*(48) = .03, *p* = .81; BF_10_ = 0.18 (see Fig. 4A).

Furthermore, we examined whether numerosity judgment performance (ratio point at 75% accuracy) predicted demand selection rates; this correlation was nonsignificant, *r*(48) = .01, *p* = .93; BF_10_ = 0.17 (see Fig. 4B).

Beyond these three correlational analyses, we performed two exploratory analyses. First, we assessed the relationship between participants’ numerosity judgment RT and their ACVS optimality rate. Here, we only consider the RT of the 2:1 ratio condition, for two reasons. First, all of our included participants performed with high accuracy at this ratio, which avoids potential impacts of a speed–accuracy trade-off; second, the numerosity judgment performed with a 2:1 ratio is closely matched to that of the ACVS task. Results showed a nonsignificant negative correlation between individual’s numerosity judgment RT and their optimality, *r*(48) = −.17, *p* = .23; BF_10_ = 0.35.

For the second exploratory analysis, we tested whether the performance difference (in both RT and accuracy) between the two word-cue search tasks predicted demand selection rates. Results revealed no significant correlations, RT difference: *r*(48) = .15, *p* = .31; BF_10_ = 0.29 (see Fig. 4C); accuracy difference: *r*(48) = −.19, *p* = .18; BF_10_ = 0.41 (see Fig. 4D).

Overall, the most notable findings from these correlational analyses is that we found no significant relationships between demand selection performance and other variables—particularly that demand selection behavior did not predict ACVS optimality. This might have been due to the extreme avoidance of numerosity judgment effort on the individual level. That is, 36 participants (72%) chose the low-demand choice in more than 90% of total trials. Therefore, this restricted range in preference for such a large group of our participants limits any potential covariance with other variables. We will revisit this issue in Experiment 2.

#### Robustness check

Since we had to exclude so many participants, we also performed an exploratory analysis to test the robustness of our main findings. By lowering the accuracy cutoff of the numerosity judgment task to .8 on the 2:1 ratio condition, data from 25 more participants were included in this exploratory analysis. All patterns of results were unchanged. In particular, the average proportion of trials on which the low-demand alternative was selected was .886 (*SD* = .197), which differed significantly from chance level (Wilcoxon signed-rank test, *p* < .001, *r* = .807). Additionally, avoidance of the numerosity judgment task was still not correlated with participants’ ACVS optimalitym *r*(73) = .04, *p* = .75.

### Discussion

In Experiment 1, we aimed to test the hypotheses that the suboptimal strategy in the ACVS task was related to (1) avoidance of cognitive effort related to the numerosity judgment process and (2) numerosity judgment ability. Here, we observed a strong preference for the color-cued search task (low demand choice) in the demand selection task on the group level. This suggests that the numerosity judgment process might pose a significant obstacle that people aim to avoid during a visual search task. However, at the individual level, such avoidance did not correlate with participants’ visual search strategy, which might be due to the ceiling effect of the extreme avoidance. Additionally, individuals’ numerosity judgment ability, as captured by their 75% accuracy thresholds in a separate task, did not correlate with their optimality either. This indicates that the observed suboptimal search behaviors were unlikely to be attributed to the numerosity judgment ability.

One key limitation of this study was that we observed significant differences in performance across two types of the cued search task, as the RT was much longer and error rates higher when performing the subset cue task. Thus, one alternative explanation for such a strong preference might be due to the avoidance of errors or the preference for a shorter task duration instead of the avoidance of related numerosity judgment effort (Kool et al., 2010). In this experiment, for each individual, the difference in the accuracy between the two tasks was not significantly correlated with the choice rate or RTs. Nevertheless, the large differences in performance may still explain the avoidance of numerosity judgment task. In Experiment 2, we aimed to control for this potential confound.

## Experiment 2

This experiment was almost identical to Experiment 1, with two main differences: We gave both types of cued search tasks a 1,000-ms preview. Since numerosity judgments might have happened concurrently with the onset of the display (Hansen et al., 2019), participants may have been sensitive to an opportunity cost between the numerosity judgment and search. Specifically, the participants may not have wanted to be estimating/comparing subsets when they could be searching instead (had they chosen the color cue task). By introducing the preview, we could expect that numerosity judgment would be completed before the search onset. This would produce more comparable RTs across the two types of cues, and it would eliminate the opportunity cost described above. At the same time, the subjective effort of having to enumerate the displays—even during the preview period—would still remain in the numerosity cue task and not the color cue task. As a result, if participants seek to avoid the effort associated with numerosity judgment, they will favor the color cue task in the demand selection. The second main difference was that we increased the sample size to obtain greater power to observe significant correlations among task measures.

### Method

#### Participants

Ninety-two individuals (44 men, 46 women, one nonbinary, one unreported) aged 19–65 years were included in Experiment 2. All participants were also recruited through the Prolific platform with the same procedure.

A similar exclusion criterion was applied here with a lowered accuracy cutoff for the numerosity judgment task to 0.8 at the 2:1 ratio. Additionally, we restricted the accuracy of both word-cue search tasks to be not lower than 0.8. An additional 73 participants were excluded from further analysis. This was due to (1) low accuracy on the ACVS task (accuracy lower than 80% or more than three standard deviations below the group average; *N* = 28); (2) low accuracy on the numerosity judgment task trials with the ratio of 1:2 (accuracy lower than 80% or more than three standard deviations below the group average; *N* = 36); (3) low accuracy on the word-cue search tasks (accuracy lower than 80% or more than three standard deviations below the group average; *N* = 9) and unusual reaction time (lower than three standard deviations below the group average; *N* = 1).

#### Stimuli and procedure

The procedure of this experiment was almost identical to Experiment 1. The main difference was that we added a 1,000-ms preview to both types of word-cue search tasks. The preview contained the word cue at the center of the stimuli and colored squares without any digits. After 1,000 ms, all digits were presented, and the search commenced. For each condition of the word-cue search task, participants first received five practice trials followed by a block of 48 trials.

### Results

#### ACVS

Mean accuracy for Standard ACVS was 96.5%. In the analysis of response time (RT), we excluded trials in which participants made an incorrect response, as well as trials with RTs less than 300 or more than three standard deviations of the mean (2.93% of the standard ACVS trials).

For Standard ACVS, mean optimality was .643 (range: .14 –.99, *SD* = .208) and mean RT was 2,907 ms (range: 1,643–4,214, *SD* = 612). RT showed a significant relationship with optimality, *r*(90) = −.55, *p* < .001.

#### Numerosity judgment

The same Weibull function was fitted measure individuals’ numerosity judgment ability. Results yielded a mean ratio of 1.27 at a 75% accuracy threshold (range: 1.08–1.56, *SD* = .11).

#### Cued search task

While the difference in RT between these two cued search tasks was reduced by the added preview, there was still a significant difference in RT between the color cue condition (*M* = 2,735 ms, *SD* = 539) and the subset cue condition (*M* = 2,979ms, *SD* = 646), *t*(91) = 5.45, *p* < .001. However, there was no significant difference in accuracy between these two conditions (color cue *M* = .958, *SD* = 0.037; subset cue *M* = .953, *SD* = 0.043), *t*(91) = 1.22, *p* = .23.

#### Demand selection task

**Fig. 5:**
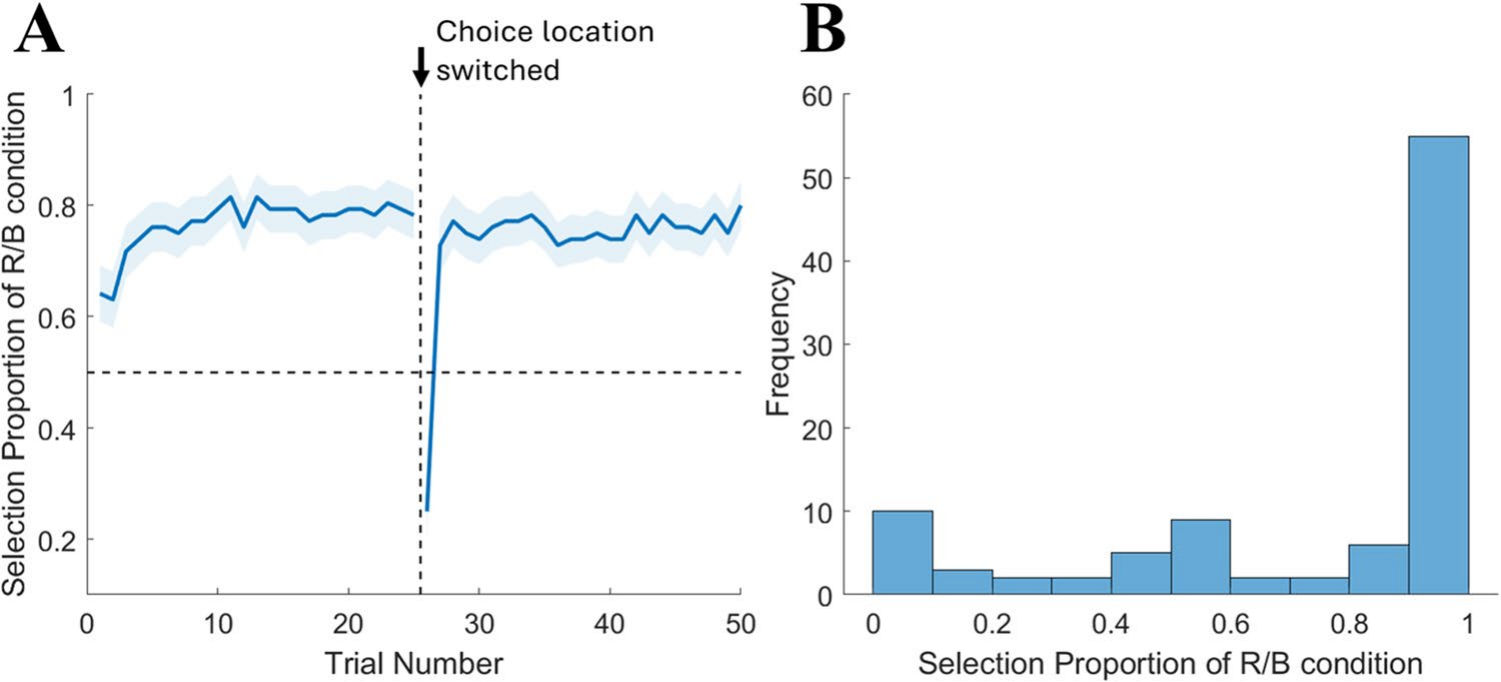
**A** Group-averaged proportion of the low-demand choice (red/blue), plotted by trial number, during the demand selection task in Experiment 2. **B** Histogram of individuals’ low-demand selection proportion in experiment 2. R/B = red/blue color cue task

The percentage choice rate of the low-demand task was .752 (*SD* = .317). Sixty-nine participants (75%) chose the low-demand choice more frequently. The overall choice rates differed significantly from the chance level (Wilcoxon signed-rank test, *p* < .001, *r* = .584). As in Experiment 1, we again observed a strong avoidance of the task associated with the high demand of numerosity judgment effort (see Fig. 5B).

#### Relationships between measures

**Fig. 6:**
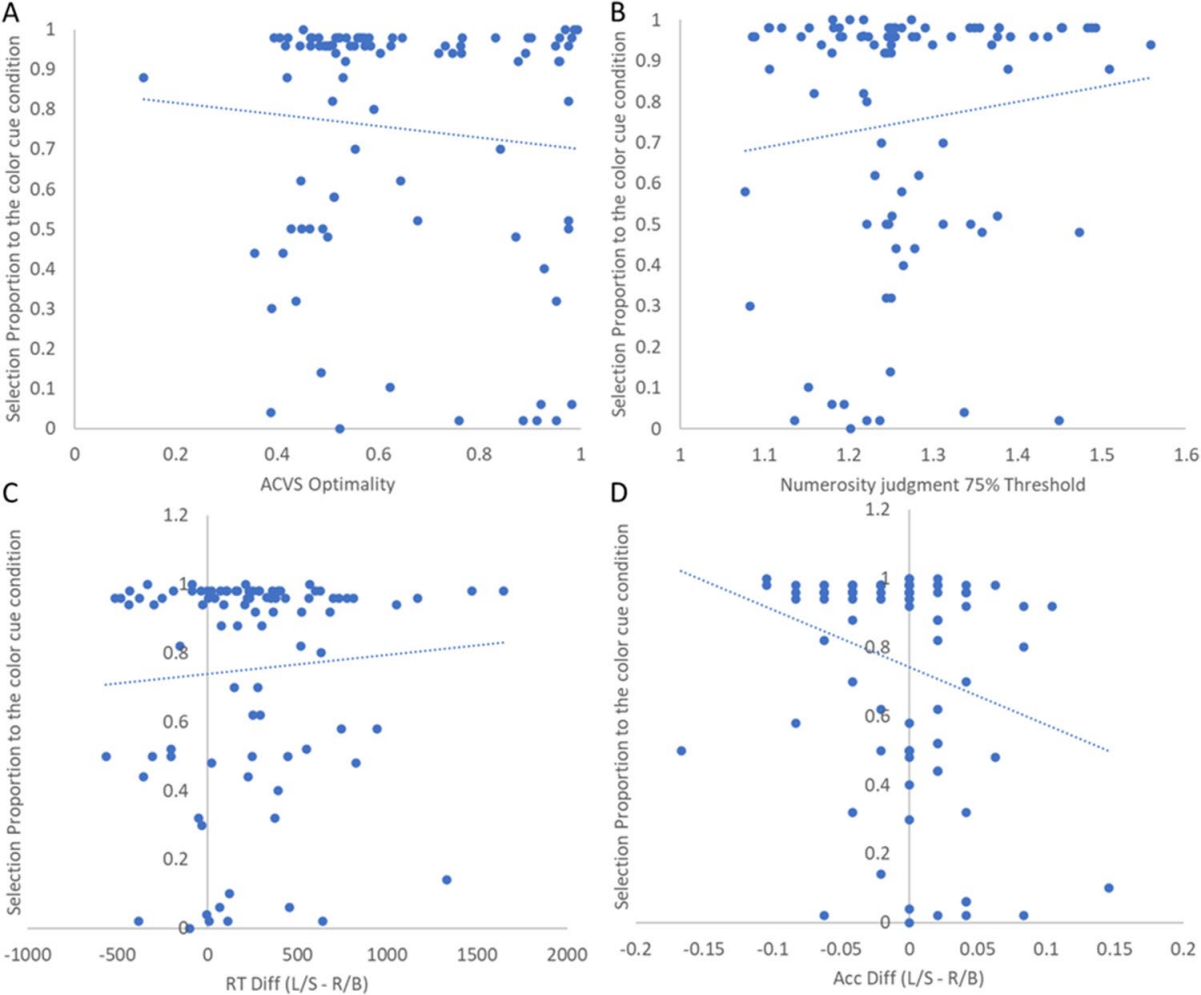
Scatter plots of correlations in Experiment 2 between (**A**) ACVS Optimality and DST selection proportion to the color cue task; (**B**) numerosity judgment ability and DST selection proportion; (**C**) RT differences between two types of word-cue search task and DST selection proportion; (**D**) accuracy differences between two types of word-cue search task and DST selection proportion

As in Experiment 1, we compared how the task measures related to one another, and we performed the same three correlational analyses. We tested the relationship between numerosity judgment ability and ACVS optimality first. Results revealed a non-significant relationship, *r*(90) = −.04, *p* = .69; BF_10_ = 0.14, consistent with our results from Experiment 1. Next, we asked whether individuals’ demand selection rates predicted ACVS optimality. There was no significant relationship between participants’ optimality and the avoidance of numerosity judgment effort, *r*(90) = .09, *p* = .37; BF_10_ = 0.19, possibly because many individuals again showed ceiling-level biases for the low-demand choice (55 participants chose the low-demand choice in more than 90% of the trials). Furthermore, we examined whether numerosity judgment performance (ratio point at 75% accuracy) predicted demand selection rates; this correlation was nonsignificant, *r*(90) = .12, *p* = .69; BF_10_ = 0.26 (see Fig. 6B).

**Fig. 7:**
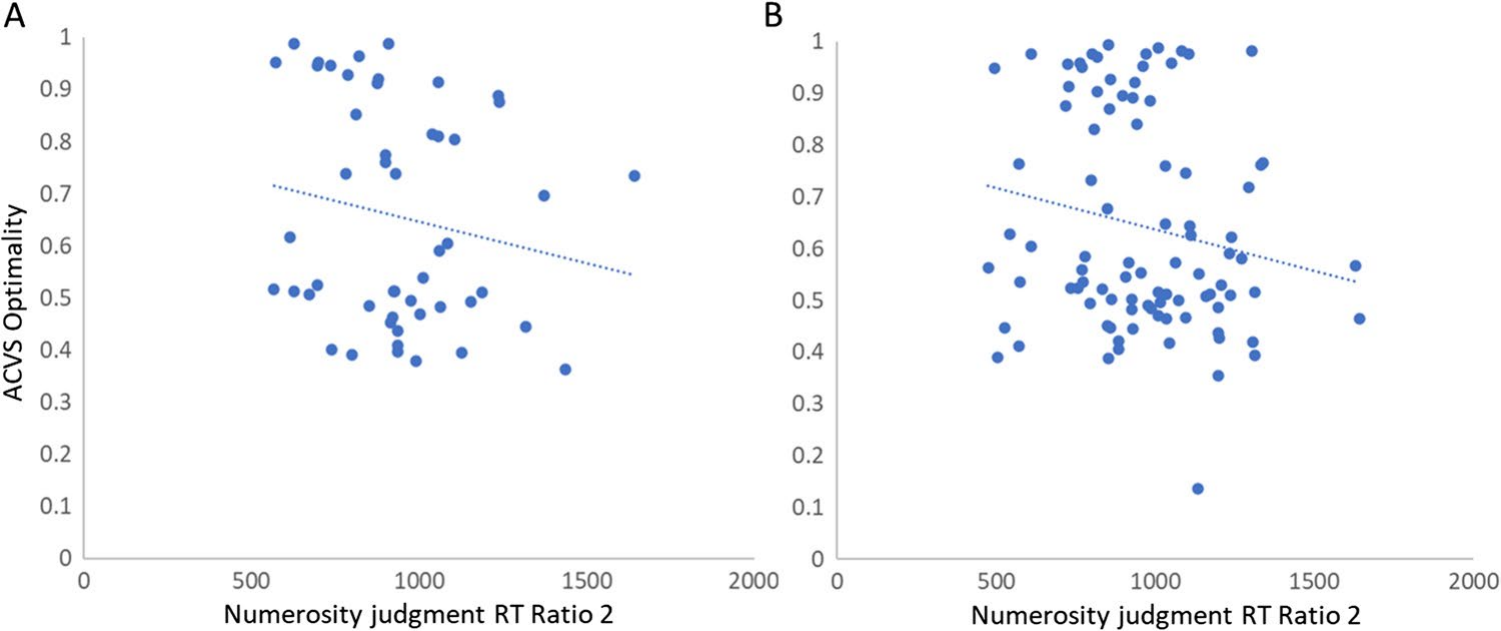
The scatter plots of numerosity judgment RT of ratio condition 2:1 and ACVS optimality for (**A**) Experiment 1 (*N* = 50); *r*(48) = −.17, *p* = .23; (**B**) Experiment 2 (*N* = 92); *r*(90) = −.184, *p* = .079

Similarly, we explored the relationship between participants’ RT when doing the numerosity judgment task ratio of 2:1 and their optimality in ACVS. Results showed a marginally significant negative correlation between individual’s numerosity judgment RT and their optimality, *r*(90) = −.184, *p* = .079 (see Fig. 7B), although the Bayesian Factor (BF = 0.59) indicated anecdotal support of the null hypothesis. We also tested whether the performance difference between the two word-cue search task predicted demand selection rates. Results revealed no significant correlations between the RT difference and demand selection rates, *r*(90) = .07, *p* = .48; BF_10_ = 0.16 (see Fig. 6C). However, we did observe a significant correlation between accuracy difference and individual’s selection rates, *r*(90) = −.25, *p* = .01; BF_10_ = 2.53 (see Fig. 6D), indicating that participants may have been sensitive to their accuracy differences in the two word cue search tasks—and that this difference impacted their demand selection.

### Discussion

In Experiment 2, we sought to control for the alternative hypothesis of Experiment 1 results that the decisions made in the demand selection task were influenced by participants’ performance such as RTs (Bogacz et al., 2006) and accuracy (Kool et al., 2010). Here, we gave both cued search tasks a preview stage to keep participants’ performance at a similar level. The preview did indeed largely reduce the performance differences between the two tasks, and the majority of participants still showed a strong preference for the color cue task, albeit to a slightly lesser extent than Experiment 1. Notably, we still observed a significant RT difference between the numerosity and color cue search tasks, but this difference did not predict individuals’ choice rate of the low-demand alternative. Conversely, while there was no significant accuracy difference between the two cued search tasks, individuals’ sensitivity to accuracy somewhat related to their demand selection choices, albeit only explaining 6% of the total variation in individuals’ selection proportion. Overall, these results support the conclusion that demand selection preference was not solely driven by the performance differences but rather the avoidance of numerosity judgment effort.

In this experiment, we also investigated whether individuals’ numerosity judgment ability influences their choice of visual search strategies. Similar to the findings in Experiment 1, we found no significant correlations between numerosity judgment ability and ACVS optimality. However, with this larger sample size, we observed a small but significant negative correlation between individuals’ RT when doing the numerosity judgment task, particularly in the condition of ratio 2:1, and their ACVS optimality. This may suggest that an individual’s choice of search strategy would be influenced by how quickly they can execute all the necessary processes involved in that strategy. If it takes too much time, even if their performance is comparable to others, individuals may still avoid using that strategy as it may confer reduced benefits.

## General discussion

Goal-directed attentional control is complex. The range of our attentional control abilities has been studied comprehensively, but strategy has been the focus of much less research. In this project, we made modifications to the Adaptive Choice Visual Search task to study people’s visual search strategies and explore why they are frequently suboptimal (Lee & Anderson, 2023). We attempted to uncover specific cognitive effort components people avoid when doing the ACVS task. Here, we focused on one such component that is essential for achieving the optimal search strategy: the numerosity judgment (Hansen et al., 2019). We used a numerosity judgment task with a similar display setting to the ACVS task to measure people’s numerosity judgment ability, and we used the demand selection task (Kool et al., 2010) to capture people’s avoidance of numerosity judgment effort. We investigated how numerosity judgment avoidance relates to visual search strategies in the ACVS task.

Our results showed that, at the group level, people did overwhelmingly avoid numerosity judgment effort. Even in Experiment 2, where participants showed largely similar performance in both cued-search tasks, most of them stuck with choosing the low-demand task choice (color-cued search task) and avoided doing the numerosity judgment when given the choice. Furthermore, such avoidance was not driven by people’s numerosity judgment ability, as individuals who were good at the numerosity judgment still showed a preference to avoid it. Our results demonstrated that the numerosity judgment process required some level of effort, leading people to tend to avoid it. However, the restricted variation in our data that was due to extreme avoidance in the DST by many participants may have limited our ability to further explore any possible relationship between effort avoidance and choice of visual search strategy (reflected in ACVS optimality) on the individual level; 36 participants (72%) chose the low-demand choice on more than 90% of total trials in Experiment 1 and 55 participants (60%) chose the low-demand choice on more than 90% of the trials in Experiment 2.

To address the issue of restricted variance and further investigate whether numerosity judgment avoidance might predict ACVS optimality, we conducted two exploratory analyses. First, we removed those individuals who predominantly chose the color cue task (more than 90% of trials) and reexamined the relationship between ACVS optimality and DST choice rate. We focused on Experiment 2 (which had a larger sample), and we were able to include 37 participants included in this analysis. We found a moderately negative, albeit nonsignificant relationship, *r*(35) = −.32, *p* = .053. The directionality of this relationship is consistent with the notion that greater demand avoidance predicts lower ACVS optimality. Second, we compared the optimality between people with a high preference (>90%) for the color cue task (*N* = 55) and people with a high preference (>90%) for the numerosity cue task (*N* = 8). Results revealed a difference in the predicted direction, although it did not reach significance (preferred color cue *M* = .65, preferred numerosity cue *M* = .79), *t*(61) = 1.89, *p* = .063, *d* = 0.71. Both of these exploratory analyses suggest a potential negative impact of the avoidance of numerosity judgment effort on participants’ search strategies in ACVS, although further, higher-powered, follow-up studies are warranted.

We also did not find a relationship between individual’s numerosity judgment ability, as derived from performance on a separate psychometric task, and their optimality in visual search. This suggests that suboptimal behavior is unlikely due to limitations in ability, such as accurately identifying the smaller color subset in our paradigm. However, in Experiment 2, we observed a weak correlation between individual’s numerosity judgment RT and their ACVS optimality. This suggests that individuals’ strategy choices may depend on their execution speed. If they are too slow, even with comparable accuracy, they might avoid the strategy, which may relate to realizing smaller gains during the ACVS task. Nonetheless, given the exploratory nature of the analysis and the inconsistency between Experiment 1 and Experiment 2, we are cautious not to overinterpret the results.

Beyond its relevance to visual search strategy, this study also offers a new insight into the process of numerosity judgment. While previous research has shown that it is highly efficient (Ariely, 2001; Chong & Treisman, 2003; Halberda et al., 2006), it is apparently not effortless. Further, this work shows that we cannot safely assume an individual will use a cognitive capacity just because they can. Research on the use of numerosity judgment to solve real world visual challenges may better quantify how frequently individuals actually make use of their robust ability to enumerate.

Although our work shows that people avoided numerosity judgment effort, we are cautious to conclude that only numerosity judgment-related effort determines strategy choice in the ACVS task. Some work in progress in our lab has found that people were inclined to use the optimal strategy when they were required to make an explicit decision regarding which color subset to search for, which potentially indicates that cognitive effort related to decision-making—not just numerosity judgment, per se—also influences strategy choice (Irons & Leber, 2018b). Furthermore, we have found that explicit instruction on the optimal strategy also increases optimality (Irons & Leber, 2018b; Zhang et al., 2024). Thus, the role of explicit knowledge appears to be important, although it is possible that avoidance of the effort required to discover more optimal strategies is a key factor.

The failure to definitively link individual differences in numerosity judgment effort with strategy choice places an important limitation on what we can conclude from this work. Unlike the individual differences we have previously observed in overall strategy choice, which were quite stable and showed high test–retest reliability (Clarke et al., 2022; Irons & Leber, 2018a), cognitive effort may be hard to quantify at the individual level. The demand selection task has shown great power in detecting effort avoidance at the group level (Kool et al., 2010; Pauszek, 2019) and revealing preference for one form of expected uncertainty over others (Gibson et al., 2021). Even more, it has shown potential to investigate individual differences in self-control costs (Kool et al., 2013). But, as mentioned in the Discussion of Experiment 1, such avoidance was so extreme in our study that we could barely observe any variance across individuals. Some argue that the design of the demand selection task creates an intense experimental situation, which strongly impacts participants’ behavior and minimizes individual variations (Strobel et al., 2020). Others have questioned whether the demand selection task is suitable for capturing general preference for cognitive effort investment at the individual level (Juvina et al., 2018), while others have suggested that with a slight modification, the demand selection task can reveal individual differences in effort discounting (Strobel et al., 2020; Westbrook et al., 2013; Westbrook et al., 2019). Thus, future work may focus more on how to quantitatively capture individual variation in effort avoidance and link it back to strategy choice. Further, when investigating the effort-based account of suboptimal visual search behavior, one should not overlook the cognitive effort carried by search itself (Anderson & Lee, 2023). Future work may pay close attention to the tradeoff between different types of effort involved in the optimal search strategy, such as numerosity judgment effort and search effort. Moreover, pupillary response has been advocated as a reliable indicator of cognitive effort involved in different control tasks (Pauszek, 2019; Porter et al., 2007; Van der Wel & Van Steenbergen, 2018), which may serve as a powerful tool for capturing effort factors at the individual level. If reliable differences in pupillary responses can be observed across multiple strategy conditions, this method may expand our understanding of people’s choices of the visual search strategy.

While the accuracy difference between the two word-cue search task types had a minor impact on participants’ choice rates in DST during Experiment 2, we did not detect any other factors influencing the selection choice rate. However, one might speculate that the cue word itself influenced participants’ choices, as the cue word “large” may be associated with a negative affective appraisal, compared with “"small.” When choosing this numerosity cue task, participants had an equal chance of receiving either cue word. Thus, this issue should theoretically be less significant as the effects of the two cues balance each other out. Nevertheless, we acknowledge that the cue words used in our experiments may have elicited negative feelings and could have exaggerated the avoidance of the numerosity cue task.

In conclusion, this study provides novel evidence that strategy use could be driven by effort avoidance. Effort in a single task is not a unitary construct. Here, we focused on the numerosity judgment effort and find that it appears to matter. These results fit parsimoniously with previous work showing that subjective evaluation of effort influences strategy use (Irons & Leber, 2018a, 2020). Additionally, these findings lay the foundation for developing an effort-based account of strategy use. Future work will further explore individual variation in how effort guides attentional control strategy.

## Supplementary information

Below is the link to the electronic supplementary material.

Supplementary file1 (DOCX 207 KB)

## Data Availability

All data are available at Open Science Framework (OSF) for all experiments (https://osf.io/2n5ma/).

## Footnotes

1 In this study, our primary focus was on the optimality measure. We also analyzed and provided data on switch rates, which represent the percentage of trials where individuals chose a different target color than on the previous trial. This information can be found in Supplemental Material Sections 1–2.

## Code availability

Experimental code are available at Open Science Framework (OSF) for all experiments (https://osf.io/2n5ma/).
