# Supplementary material for: Investigating an effort avoidance account of attentional strategy choice
Source: Atten Percept Psychophys. 2024 Jul 26;86(6):1989–2002. doi: 10.3758/s13414-024-02927-1 (PMC11411006; doi:10.3758/s13414-024-02927-1)
Supplement: Supplementary file 1 — Supplementary file1 (DOCX 207 KB) [file 13414_2024_2927_MOESM1_ESM.docx]

**Supplementary Materials**

1. Switch rate analyses

We also looked at a secondary measure, switch rate, or the percent of trials in which individuals chose a different target color to the target on the previous trial. While switch rate is to some extent dependent on optimality rate (choosing the optimal target on every trial requires switching targets whenever the smallest subset color changes across trials), unnecessary switching often has been associated with slowed RTs in our preview studies (Irons & Leber, 2016; 2018).

The results of switch rates are reported here. In Exp 1, switch rate ranged from .197 to .519 (M = .358, SD = .081), and it did not significantly correlate with selection proportion to the low demand choice (color-cue search condition; r(48) = .103, p = .48; see figure 1A). In Exp 2, switch rate ranged from 0 to .58 (M = .359, SD = .127) and it did not significantly correlate with selection proportion to the low demand choice (color-cue search condition; r(90) = .097, p = .36; see figure 1B).


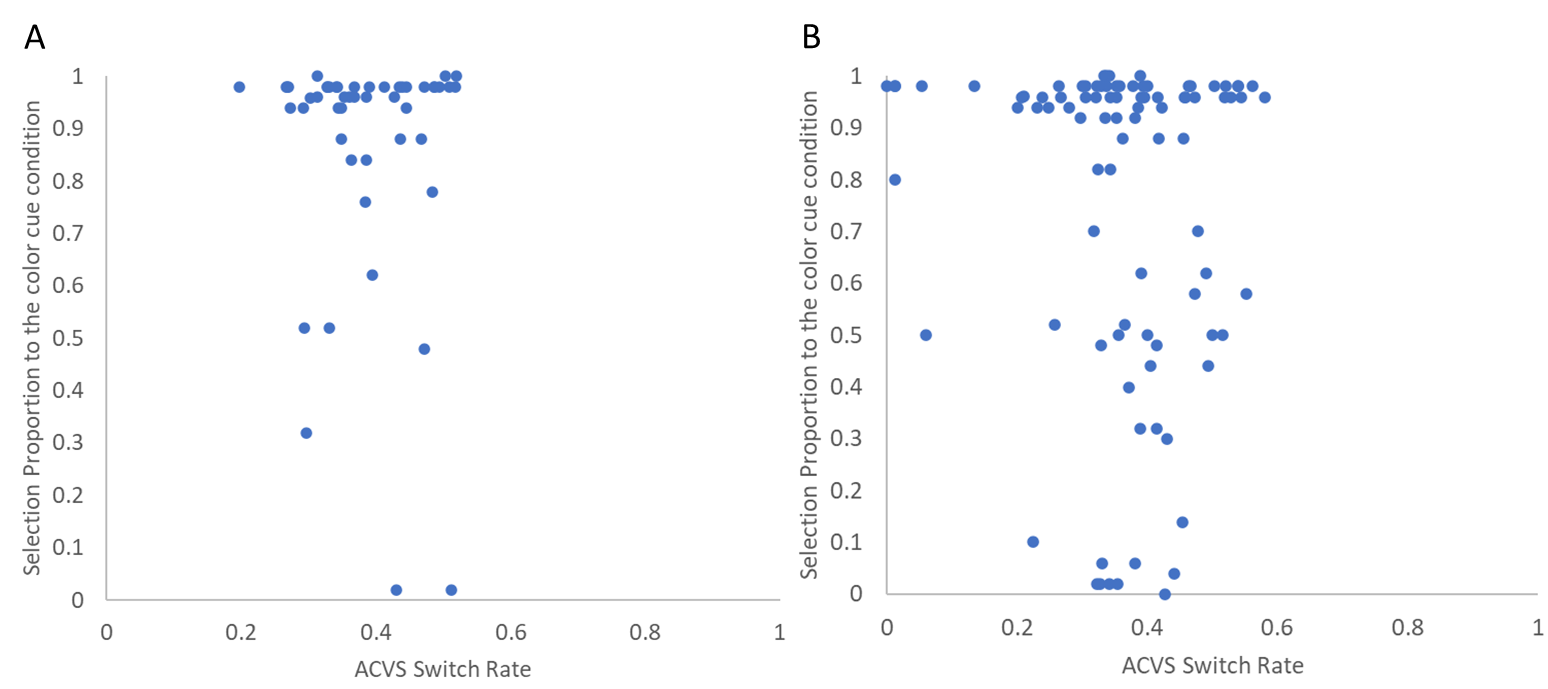


*Supplementary Figure 1*. Scatter plots for ACVS Switch rates and Selection proportion to the color cue condition in DST. A) Exp 1; B) Exp 2

1. Relationship between switch rate and optimality

Here, we provide scatter plots illustrating the relationship between optimality and switch rate (see Figure 2). The observed suboptimal search behaviors can be predominantly attributed to two factors: excessively frequent switching of the reported target color (~50%, considerably higher than the rate required for the optimal strategy, ~33.3%) or a strong tendency to concentrate on one target color without switching. These results are consistent with our earlier studies and show the characteristic pattern of decreasing variation as optimality increases, since switching must converge at a precise rate (in this study, ~33.3%) to produce 100% optimality (e.g., Irons & Leber, 2016*;* 2018).


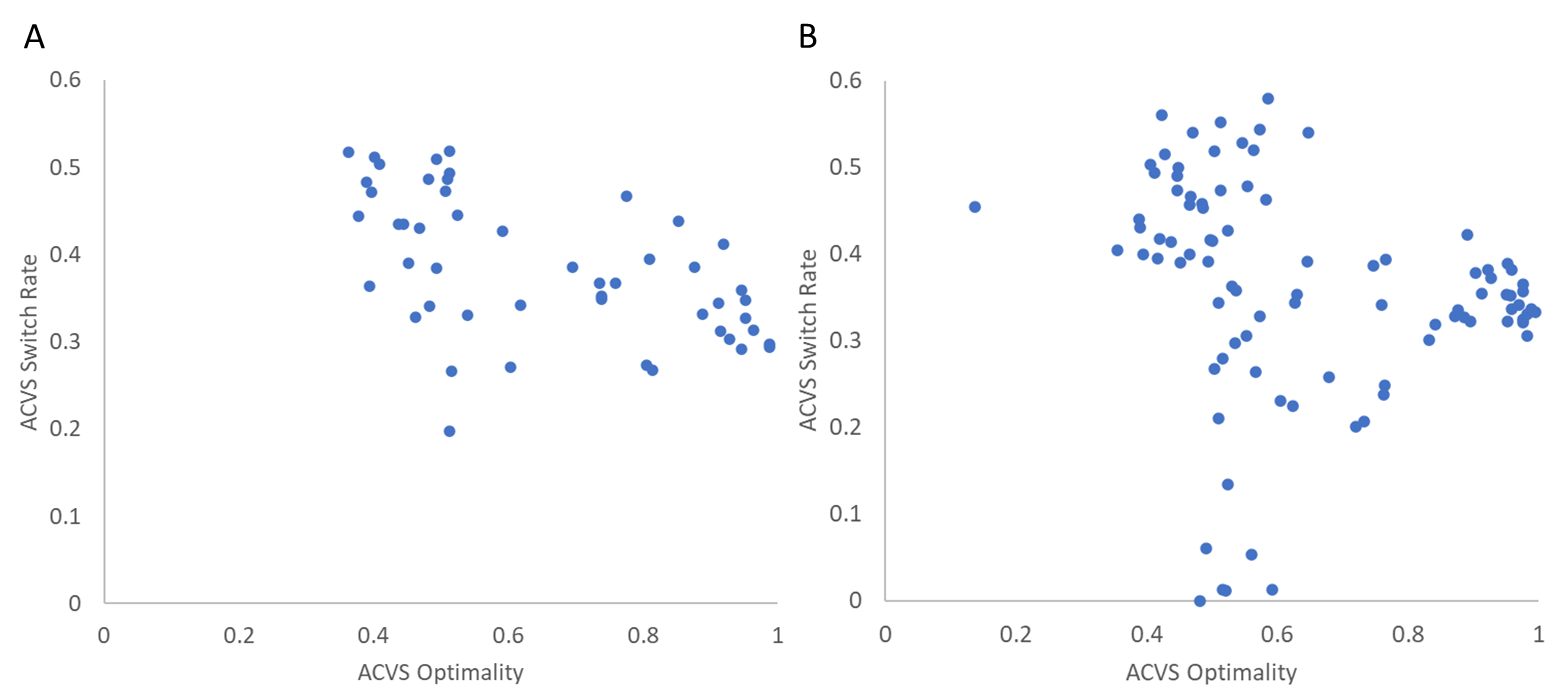


*Supplementary Figure 2*. Scatter plots for ACVS Optimality and Switch Rate from A) Exp 1;B) Exp 2
